# Screening for comprehensive social needs in patients with cancer: a narrative review

**DOI:** 10.1093/jncics/pkaf012

**Published:** 2025-01-28

**Authors:** Isabel Arana, Raymond Liu, Lawrence Kushi, Erin Hahn, Meera Ragavan

**Affiliations:** Kaiser Permanente Bernard J. Tyson School of Medicine, Pasadena, CA 91101, United States; Department of Hematology/Oncology, Kaiser Permanente, San Francisco Medical Center, San Francisco, CA 94143, United States; Division of Research, Kaiser Permanente Northern California, Pleasanton, CA 94566, United States; Department of Research and Evaluation, Kaiser Permanente Southern California, Pasadena, CA 91101, United States; Department of Hematology/Oncology, Kaiser Permanente, San Francisco Medical Center, San Francisco, CA 94143, United States; Division of Research, Kaiser Permanente Northern California, Pleasanton, CA 94566, United States

## Abstract

**Background:**

Patients with cancer who report social needs have worse quality of life, lower health-care access, and suboptimal health outcomes. However, screening for social needs does not happen systematically, and successful screening tools, strategies, and workflows have seldom been described. The downstream effects of screening including resource navigation have also not been well characterized. The objective of this narrative review was to fill these gaps.

**Methods:**

Two investigators searched PubMed and Embase for studies that implemented a patient-facing social screening tool among patients with cancer between 2008 and 2023 using search terms including *social screening*, *social*  *needs*, and *cancer*.

**Results:**

We identified 19 articles that met study inclusion criteria. The most common tool used was the validated Health Leads Social Toolkit. Most often, screening tools were administered electronically, sent directly to patients, and captured needs at a single time point during a patient’s diagnosis. Screening response rates ranged between 10% and 60%. Less than half of the studies described downstream resource navigation for patients who screened positive for social needs. Only 1 study evaluated the impact of screening on clinical outcomes and quality of life. Screening for patients who do not speak English or who belong to historically racial, ethnic, and gender minority groups was limited.

**Conclusions:**

Screening for social needs has been shown to be feasible across delivery systems with numerous validated tools available. However, gaps remain in generalizability to diverse patient populations. Future work must identify how screening workflows can be successfully incorporated into routine clinical workflows.

## Introduction

Patients with cancer often face social needs that impede access to high-quality, timely, and equitable health care.^1-3^ These needs span several social domains or categories such as housing,^4^^,^^5^ transportation,^6^^,^^7^ social isolation,^8^ financial challenges,^9^^,^^10^ and food security.^11^^,^^12^ Social needs are associated with suboptimal clinical outcomes across the cancer continuum including delayed diagnoses, lower receipt of guideline-concordant care, decreased access to clinical trials, and higher overall mortality rates.^4^^,^^6^^,^^13^ In addition, patients with social needs report lower health-related quality of life and psychosocial functioning.^14-16^

Several validated tools exist to capture social needs in health-care settings, albeit not specific to patients with cancer. These include the 10-item Accountable Health Communities Screening Tool developed by the Center for Medicare and Medicaid Innovation; the Protocol for Responding to and Assessing Patients’ Assets, Risks, and Experiences; and the Health Leads Toolkit.^17-19^ Despite the well-described impact of social needs on cancer outcomes, patients are not screened for such needs in a systematic way in the United States.^20^ Screening for social needs can facilitate resource navigation early in a cancer diagnosis by which patients are connected to social workers or care navigators or are directly referred to local or national resources that can mitigate needs.^21^ In addition, social needs screening can help clinicians tailor treatment strategies appropriately. Examples include extended chemotherapy dosing intervals for a patient who faces transportation challenges^22^ or early referrals to government-funded food assistance programs for patients with food access concerns.^23^ Implementation of feasible, effective, and sustainable multidomain social needs screening programs is critically needed. However, effective strategies are lacking.

In this narrative review, we sought to (1) characterize which social needs screening tools have been tested and implemented in cancer care delivery settings, (2) describe the implementation of social needs screening, and (3) evaluate the impact of social needs screening on psychosocial and clinical outcomes.

## Methods

We performed a literature search using PubMed and Embase. We conducted 2 identical searches in each database using 2 search strategies. The first strategy comprised keywords *social needs* AND *cancer*, and the second strategy comprised keywords *social screening* AND *cancer*. We picked these terms based on consensus from members of the study team, all of whom have expertise in health disparities. We excluded studies published more than 15 years ago out of concern for lack of relevance. We included studies conducted in the United States between January 1, 2008, and June 30, 2024, that included social needs screening across 2 or more domains for patients with cancer. We excluded studies conducted outside the United States, studies that did not address patients with cancer, and studies that did not implement patient-facing social needs screening tools (eg, studies that used traditional demographic variables, such as annual household income, to assess social risk). Two members of the study team conducted each search and cross-checked all studies to confirm that the studies were appropriate for study inclusion.

## Results

**Figure 1.**
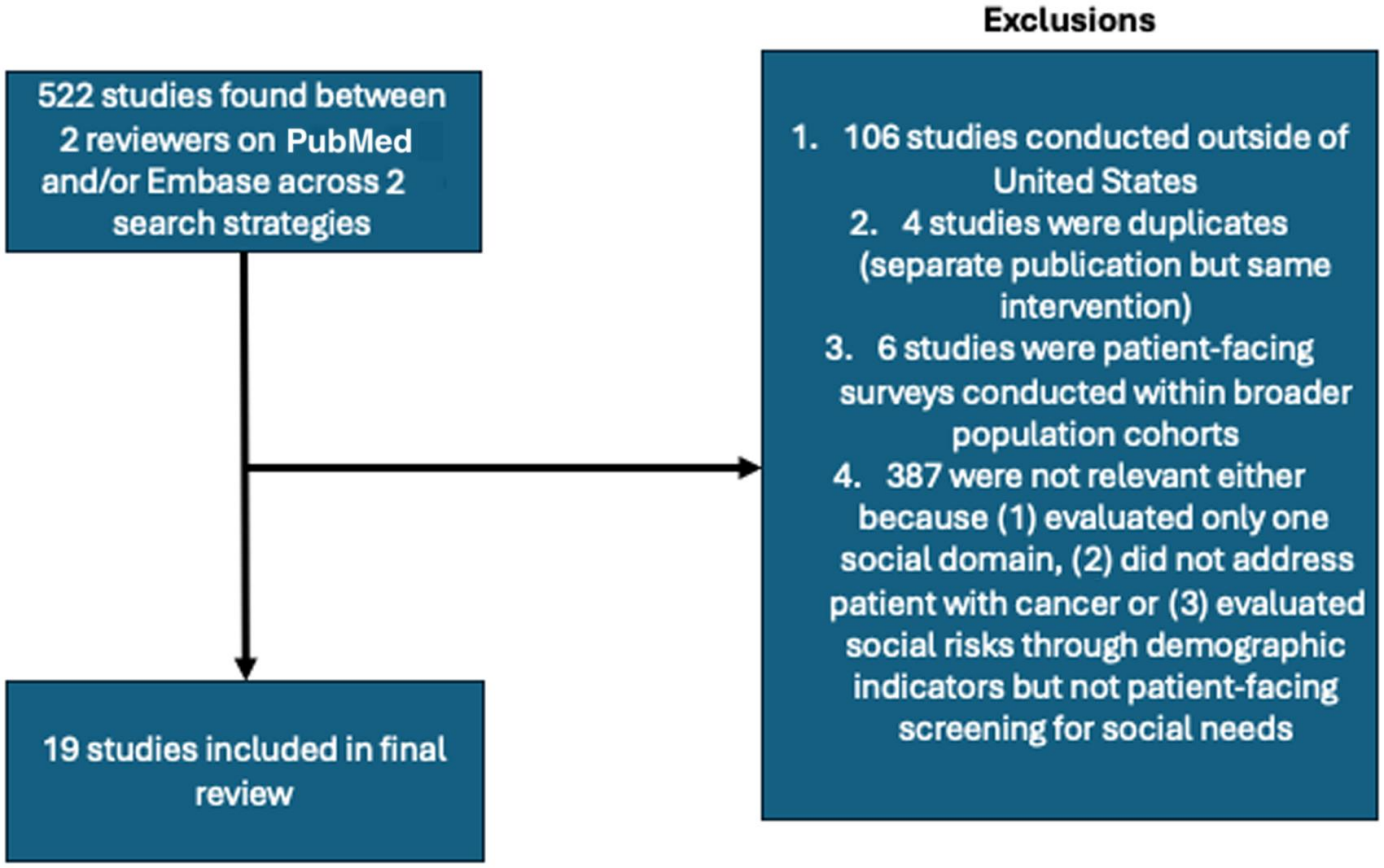
Consort diagram describing study inclusion.

A total of 522 studies were found across PubMed and Embase, of which 503 did not meet study inclusion criteria (Figure 1). A total of 19 studies were included in the final review. Most studies were single center (n = 12) and evaluated screening at a single time point (n = 20).

### Screening tools

Screening tools ranged from validated instruments to novel tools developed or adapted by study investigators (Table 1). The most frequently used validated tool was the Health Leads Social Needs Screening Toolkit.^1^^,^^15^^,^^28^^,^^34-37^

**Table 1.** List of established social screening tools used across studies

Validated	Domains analyzed	Description	Reference link	Studies for which tool was used
Tool				
Health Leads Social Toolkit	Housing instability, food insecurity, transportation problems, utility help needs, financial resource strain, exposure to violence	A 7-item validated survey developed by Health Leads, a nonprofit organization with the mission to advance health equity	https://healthleadsusa.org/news-resources/the-health-leads-screening-toolkit/	^24^
Accountable Health Communities-Health-related Social Needs Tool	Housing instability, food insecurity, transportation problems, utility help needs, interpersonal safety	A 26-item validated survey developed by Centers for Medicare & Medicaid Services Center for Medicare and Medicaid Innovation	https://www.cms.gov/priorities/innovation/files/worksheets/ahcm-screeningtool.pdf	^25^
Functional Assessment of Cancer Therapy-General	Physical, social or family, emotional, and functional well-being domains	A 27-item validated survey that measures health-related quality of life in cancer patients	https://www.facit.org/measures/fact-g	^26^
Psychosocial Assessment Tool	Family structure and resources, family problems, social support, child problems, acute stress, sibling problems	Validated psychosocial risk screening tool based on the Pediatric Psychosocial Preventative Health Model	https://www.psychosocialassessmenttool.org/	^27^
COST tool	Financial concerns, job security	A 12-item survey developed in conjunction with the University of Chicago that measures financial distress experienced by cancer patients	https://www.facit.org/measures/facit-cost	^14^ ^,^ ^28^
Protocol for Responding to and Assessing Patients’ Assets, Risks, and Experience	Housing, insurance, work security, money, resources, utilities, stress, social and emotional health	A 21-core-item survey designed through stakeholder engagement, paired with an Implementation and Action Toolkit, and standardized across Internal Classification of Disease (ICD)-10, Logical Observation Identifiers, Names, and Codes (LOINC), and Systematized Nomenclature of Medicine (SNOMED)	https://prapare.org/the-prapare-screening-tool/	^29^
Needs Assessment for Advanced Lung Cancer Patients	Psychological and emotional, financial, social, spiritual, symptom, daily living, and medical communication	A 38-item survey across 7 domains adapted from the larger Needs Assessment for Advanced Care Patients survey	https://pubmed.ncbi.nlm.nih.gov/22499399/	^30^
Innovative Screening Tools				
Social Work Assessment Tool	Financial resources, safety issues, comfort issues, social support, caregivers, loss of income, anxiety about death	A 1-page guide across 11 social domains created specifically for social workers developed by the National Hospice and Palliative Care Organization’s Council of Hospice and Palliative Professionals	http://www.kvccdocs.com/KVCC/2016-Spring/MHT216/lessons/L-25/SW%20End%20of%20Life%20Assessment.pdf	^27^
Health disparities survey based on Warnecke’s et al. conceptual model^31^	Patient demographics including self-reported race and ethnicity, acculturation, health literacy, perceived discrimination, medical mistrust, and neighborhood perceptions	Details of survey not provided by investigators	N/A	^32^
Patient-facing mobile application	Finance, food, utilities, housing, transportation, social, hardship	A 36-item survey across 7 domains; specific survey questions not provided	N/A	^33^
Single question prompt for social needs	Housing, food, transportation, medication affordability	“Is the amount of income you have available in a typical month not enough for any of the following needs?”	N/A	^28^

### Screening modalities

Studies used various modalities to implement tools, including emailing or messaging the tool to the patient online,^14^^,^^15^^,^^35^^,^^38^^,^^39^ screening via a research or health-care staff member on the phone or in-person,^15^^,^^25^^,^^26^^,^^33^^,^^40^^,^^41^ or point-of-care tools given to patients to complete during visits to the health-care facility.^26^^,^^28^^,^^30^^,^^31^^,^^34^^,^^35^^,^^40^ Nearly half of all studies deployed a research team to conduct social needs screening.^14^^,^^24^^,^^26^^,^^28^^,^^32^^,^^34-36^ Some studies conducted multiple screenings throughout a diagnosis (maximum number of screens per patient was 4),^25^^,^^26^^,^^28^^,^^30^^,^^31^^,^^35^ and the remaining studies performed the screen at a single time point. A positive screen was defined by studies as the patient reporting at least 1 social need.

Studies that had at least a during-visit option for screening had the highest response rates.^34^^,^^36^^,^^42^ Some studies used during-visit and online portal–based screens and found high response rates as well.^28^^,^^34^^,^^36^ For example, Wethington et al.^34^ implemented a social needs screening as part of a quality improvement project among patients with gynecologic cancers in a single tertiary care center, employing paper-based screens (during clinic visits) and online portal–based screening. The authors found response rates improved clinically significantly when incorporating an electronic health record and patient portal–based screens (97% response rate) compared with paper-based screens alone (52% response rate).

### Populations screened

Studies varied in inclusion of non-English speaking patients and diverse patient populations. Three studies evaluated screening interventions specifically designed to target patients of minoritized backgrounds.^15^^,^^26^^,^^43^ Eight studies were conducted among patients who did not speak English as a first language and/or included a screening tool in languages other than English.^1^^,^^24-26^^,^^28^^,^^30^^,^^34^^,^^37^^,^^41^^,^^50^

### Measuring uptake

The average response rates for completing the screenings ranged from 40% to 60%, with only 1 study reporting a response rate lower than 40%.^44^

Patient or provider feedback on the screening process was collected either through surveys or interviews and used this feedback to iterate the screening process.^21^^,^^24^^,^^26^^,^^32^^,^^34^^,^^41^ One pilot study using a 2-part, 19-item survey tool found that slightly more than half of patients found the tool usable, and the majority (63%) felt comfortable answering the questions.^29^

### Social domains and resource navigation

The proportion of patients with a positive needs screen ranged from 25% to 70%. Common social needs included financial burdens, social support, psychosocial needs, and transportation. Some studies additionally described resource navigation workflows for patients who had a positive needs screen (Table 2).^21^^,^^26^^,^^28^^,^^31^^,^^34^^,^^35^^,^^37^^,^^50^ Cotangco et al.^45^ screened 1010 patients with gynecologic cancers over 9 months at a safety net hospital; 54% screened positive and were referred to a social worker or cancer care navigator, and the majority (78%) of patients referred received a list of resources. Beavis et al. found 36% of 373 screened patients had at least 1 social need, and 13% asked for a referral.^24^ The investigators leveraged the nonprofit organization Health Leads for resource navigation and found that, of the small cohort (n = 25) who were successfully referred, a majority (n = 17, 68%) had their needs resolved.

**Table 2.** Description of studies found

Reference no and author and year	Tool	Modality	Population/languages	Implementation	Response rate	Resource navigation	Limitations
Coughlin et al., 2022^44^	Health Leads	Paper survey by mail	Hematologic cancer survivors treated at a single center in Georgia Languages not described	Mailing supplies, accessing electronic health records for mailing addresses, manual review of responses	10.6%	None	Low response rates and selection bias
Goel et al., 2024^1^	Health Leads	Not reported	Patients with any stage of invasive breast cancer English and Spanish included	Not specified	67%	None	Screening implementation not described, COVID pandemic may have influenced responses
Hsu et al., 2024^14^	Adapted survey from the Mayo Survey Research Center, Comprehensive Score for Financial Toxicity (COST) tool, and European Organization for Research and Treatment of Cancer (EORTC) quality-of-life survey	Emailed to consenting patients in REDCap	Lung cancer survivors at least 1 year out from diagnosis seen at Johns Hopkins English only	Screening for eligible patients, telephone consent, individual emails to patients	65%	None	Not generalizable outside of research settings, conducted during COVID pandemic, which may have influenced responses
Hastert et al., 2021^15^	Health Leads	Qualtrics survey, over telephone or mailed survey	African American cancer survivors recruited through Detroit Research on Cancer Survivors cohort study Language not specified	Needs screening was part of enrollment for broader cohort study	N/A	None	Limited generalizability as took place within a broader population cohort study; recall bias
Cotangco et al., 2023^45^	Health Leads	Front desk staff administered paper survey during clinic visit	Patients with a gynecologic cancer seen in a clinic in Los Angeles English and Spanish	Front desk staff involvement, social workers	58%	Referral to cancer care navigators	Narrow demographic population, low response rate, exclusion of telehealth patients
Davis et al., 2021^29^	Adapted from AHC-HRSN and PRAPARE	Not reported	Adult patients with cancer treated within University of Pennsylvania Health System undergoing active treatment Language not specified	Not reported	44%	None	Single geographic area, implementation details not provided
Suh et al., 2018^27^	Adapted from multiple tools including psychosocial assessment tool and Social Work Assessment Tool (SWAT)	Not reported	Parents of children diagnosed with cancer across 7 hospitals in Chicago, Illinois English and Spanish	Not reported	Not reported	None	Implementation details not provided, nonvalidated survey
Wethington et al., 2024^34^	Health Leads	Both patient portal–based screen (previsit) and paper (during visit)	Patients seen at a gynecologic oncology clinic at Johns Hopkins University Specific languages not specified but authors state non-English patients were reached	Research staff and community organization partner, programmer to facilitate portal-based screens	52% round 1, 90% round 2	Referral to health coordinator	Dependence on research team for workflow, limited generalizability to other populations
Patel et al., 2021^26^	Functional Assessment of Cancer Therapy - General (FACT-G)	During clinic visit (administered by a community health worker)	Patients enrolled in broader CHW intervention in Atlantic City, New Jersey, and Chicago, Illinois Language not specified	Community health workers and community organization partners, research team	N/A	Resource navigation by CHW	Only generalizable in context of CHW intervention, cannot isolate social needs screening from other components of intervention
Neparidze et al., 2024^25^	Accountable Health Communities Survey	During visit by research staff	Patients with multiple myeloma recruited through national Patient Engagement Research Council Language not specified, but results report some patients spoke language other than English at home	Research staff, relationship to patient research council, time to conduct interviews	84%	None	Small sample size, not generalizable methods
Nyakudarika et al., 2021^36^	Health Leads	Survey administered during clinic visit or over the phone in between visits	Patients with gynecologic cancers treated at a single institution English and Spanish	Research team	N/A	Referral to psychiatrist and social worker	No longitudinal screening, narrow patient population
Beauchemin et al., 2024^35^	Semistructured interviews	Interviews with patients	Adolescents and young adults within 2 years of a cancer diagnosis English and Spanish	Research team, time to conduct interviews	N/A	None	Interview modality for screening not scalable
Mazor et al., 2022^30^	Needs Assessment for Advanced Cancer Patients (NA-ALCP)	In-person or phone by research staff	Patients with newly diagnosed advanced lung cancer English and Spanish	Pathology registry and data queries, research staff to administer screening	Not included	None	Mostly female participants, small sample size, narrow patient population
Oyedele et al., 2023^33^	Nonvalidated survey adapted from Patient Reported Outcomes Measurement Information System (PROMIS) and PhenX Toolkits	Mobile phone–based instrument	Patients and caregivers treated at a single cancer center in Baltimore, Maryland English only	Mobile app development	N/A	Referral to social worker	Single institution, lack of diversity in patient population
Beavis et al., 2020^24^	Health Leads	During-visit paper survey administered by front desk staff	Patients seen at a gynecologic oncology clinic Language not specified, but 96% of patients reported English as first language	Staffing support for screening and review of responses, collaboration with Health Leads organization	47%	Referral to social worker and Health Leads program	Did not track patients who declined to complete survey, single center, narrow patient population
Schoenberger et al., 2023^32^	Nonvalidated social needs survey adapted from health disparities conceptual model^31^	During-visit paper survey or phone survey	Patients with newly diagnosed hepatocellular carcinoma across 4 large health systems in Florida and Texas English and Spanish	Research staff to administer survey	59.6%	None	Response bias, implementation details lacking, single institution
Kronfli et al., 2022^42^	Nonvalidated survey adapted from other published tools	During-visit survey, could be returned in person or by mail	Patients undergoing curative radiation therapy across multiple cancer types English only	Resources to administer paper survey, receipt of mailed surveys, REDCap and staff to enter data	83%	Referral to social worker (patient asked as part of survey)	Longitudinal analysis not feasible, nonvalidated survey
Baughman et al., 2017^43^	None	Interviews	Patients with colorectal cancer identifying as LGBTQ	Time and staff required for interviews and coding of responses	N/A	None	Lack of generalizability to clinical settings
Thom et al., 2024^28^	COST tool and additional single-question, nonvalidated prompt for other social needs	Portal-based survey and during visit via tablet device	Patients with breast, gastrointestinal, and gynecologic and thoracic cancers	Research staff to administer survey	54%	Patients asked if they would like assistance and if so referred to institution’s financial assistance team	Single center, lack of follow-up of resource navigation

### Outcomes

Only 1 randomized study evaluated the impact of social needs screening on clinical outcomes and quality of life. Patel et al.^26^ found that a community health worker navigation program, which included social needs screening and resource navigation, reduced health-care costs, improved quality of life, and decreased hospital utilization. Of note, the impact measured in this study was of the entire community health worker intervention and not just the social needs screening.

## Discussion

In this narrative review, we found that cancer delivery systems leveraged a number of social needs screening tools, with Health Leads being the most common, and that screening modalities ranged from online or patient portal–based surveys to during-visit point-of-care screening. Most studies found that these ranges of screening methods were feasible, with response rates above 40%. However, the studies highlight a few key gaps in efforts to implement widespread social needs screening for patients with cancer. First, only a minority of studies described downstream workflows of a positive screen, including referrals to health-care staff and resource navigation. Furthermore, studies that did describe a resource navigation workflow provided only limited details on resources received by patients, a crucial area that requires ongoing attention. Second, only 1 study evaluated the impact of social needs screening on patient-reported quality of life and clinical outcomes, and this was in the context of a larger community health worker intervention. Third, although screening was found to be feasible with reasonable response rates, studies frequently reported screening workflows that required involvement from research staff and were implemented over a limited time period. Fourth, more than half of studies included only English-speaking patients, and only a few studies targeted their screening interventions toward capturing needs of minoritized patients. Strategies to make social needs screening generalizable, longitudinal, equitable, and cost saving are critically lacking.

Implementation of social needs screening into routine clinical workflows is a pressing challenge for health-care systems as payors begin to implement screening requirements. Medicare requires screening for 5 domains in inpatient settings,^46^ and the Healthcare Effectiveness Data and Information Set used by the majority of insurers to track quality at the provider level includes a 3-item social needs screen.^47^ However, needs vary depending on patient demographics and delivery settings; thus, there is unlikely to be a one-size-fits-all approach.

Effective screening and resource navigation programs will almost definitively require staffing support and time. A key challenge remains in funding for such efforts, highlighted by the studies reported in this review that leveraged research staff and funding to implement screening rather than routine clinical workflows. Medicare now reimburses for cancer navigation services that may naturally pair with social needs screening and provide a reimbursable avenue for screening, but challenges, including high staff attrition,^21^^,^^38^ remain in widespread implementation. Efforts to identify the impact of social needs screening on health-care utilization and outcomes should be accelerated to demonstrate the potential downstream cost savings of screening, such as the potential for averted emergency room visits or fewer missed appointments. These cost savings could help make institutions and payors more willing to invest in resources required for screening.

There are potential levers of action that health systems can take to move toward a model of universal social needs screening. First, there is value in designing short-term pilot studies to understand the social needs patients face, which, as our review found, will vary based on patient demographics, geographic catchment areas, payor mixes, and delivery settings. A 1000-foot view of the needs of a specific population can help health-care teams develop a resource navigation workflow and identify gaps in patients who may be at high risk for social needs, leveraging the existing health-care staff and local and community resources. Second, researchers and operational leaders should quantify potential cost savings of social needs screening and resource navigation, which in turn should be invested into additional staffing support and navigation. Staffing support should specifically target patients at high risk for suboptimal clinical outcomes, such as those belonging to racial, ethnic, or gender minority groups or patients who do not speak English as a first language and thus who may not be captured through certain screening modalities (such as patient portal–based surveys).^40^^,^^48^ Third, given the staffing challenges faced by all health systems, novel methods to capture social risk screening such as natural language processing to abstract specific elements from the electronic health record may help identify high-risk populations and obviate the need for ongoing routine screening.^39^ Although validated tools such as the Health Leads Toolkit may be preferred for comprehensive screening and are available in multiple languages allowing for equitable screening, a single-question screener (such as that deployed by Thom et al.^28^) may be a more feasible initial method to identify at-risk patients. Fourth, delivery systems should leverage third-party organizations who specialize in community resources to circumvent the limitations of clinic-level staffing. Many cancer foundations have free resource navigation services, including Health Leads,^17^ the American Cancer Society, and disease-specific organizations such as the Leukemia and Lymphoma Society.^37^ Multilevel interventions must be developed with underrepresented patient populations at the forefront and tested rigorously across multiple types of cancer settings. Hybrid implementation designs that identify feasibility and efficacy of a screening tool can help fill this important gap.^41^

Our study must be interpreted in context of limitations. We may not have captured studies that may have used broader patient-reported outcome instruments (eg, National Comprehensive Cancer Network Distress Thermometer) that encompass but do not solely report social needs. We did not include studies that leveraged national population cohorts to measure social determinants as our aim was to capture system-level screening. As a consequence, most studies we found were single center, and thus, comparisons across studies, with varying patient populations, must be interpreted with caution.

In conclusion, screening for social needs is feasible across patient demographics and delivery systems. Optimal methods for screening will vary. However, operational challenges remain in ensuring screening is equitable and sustainable outside of research settings. Such challenges require the direct effort of health-care leadership, payors, and policy makers. Future studies should evaluate the potential for cost savings and improved clinical outcomes with social needs screening.

## Data Availability

No data were generated or analyzed for this manuscript.

## References

[1] Goel N , Lubarsky M, Hernandez AE, et al Unmet social needs and breast cancer screening utilization and stage at presentation. JAMA Netw. Open. 2024;7:e2355301. 38353954 10.1001/jamanetworkopen.2023.55301 PMC10867685

[2] Graboyes EM , Lee SC, Lindau ST, et al Interventions addressing health-related social needs among patients with cancer. J Natl Cancer Inst 2024;116:497-505. 38175791 10.1093/jnci/djad269 PMC11494469

[3] National Comprehensive Cancer Network. Measuring and addressing health-related social needs in cancer: working group recommendations. 2023. Accessed June, 15, 2024. https://www.nccn.org/docs/default-source/oncology-policy-program/hrsn-wg-recommendations.pdf?sfvrsn=444faf60_4

[4] Fan Q , Keene DE, Banegas MP, et al Housing insecurity among patients with cancer. J Natl Cancer Inst 2022;114:1584-1592. 36130291 10.1093/jnci/djac136 PMC9949594

[5] Coughlin SS , Datta B. Housing insecurity among cancer survivors: results from the 2017 behavioral risk factor surveillance system survey. J. Cancer Policy. 2022;31:100320. 35559872 10.1016/j.jcpo.2021.100320

[6] Jiang C , Yabroff KR, Deng L, et al Transportation barriers, emergency room use, and mortality risk among US adults by cancer history. J Natl Cancer Inst. 2023;115:815-821. 37185777 10.1093/jnci/djad050 PMC10323887

[7] Pringle S , Ko EM, Doherty M, Smith AJB. Addressing transportation barriers in oncology: existing programs and new solutions. Support. Care Cancer. 2024;32:317. 38684580 10.1007/s00520-024-08514-2 PMC11058971

[8] Fox RS , Armstrong GE, Gaumond JS, et al Social isolation and social connectedness among young adult cancer survivors: a systematic review. Cancer. 2023;129:2946-2965. 37489837 10.1002/cncr.34934 PMC10584376

[9] Carrera PM , Kantarjian HM, Blinder VS. The financial burden and distress of patients with cancer: understanding and stepping-up action on the financial toxicity of cancer treatment. CA Cancer J Clin 2018;68:153-165. 29338071 10.3322/caac.21443 PMC6652174

[10] Narang AK , Nicholas LH. Out-of-pocket spending and financial burden among Medicare beneficiaries with cancer. JAMA Oncol. 2017;3:757-765. 27893028 10.1001/jamaoncol.2016.4865 PMC5441971

[11] Gany F , Lee T, Ramirez J, et al Do our patients have enough to eat? Food insecurity among urban low-income cancer patients. J. Health Care Poor Underserved. 2014;25:1153-1168. 25130231 10.1353/hpu.2014.0145 PMC4849892

[12] Raber M , Jackson A, Basen-Engquist K, et al Food insecurity among people with cancer: nutritional needs as an essential component of care. J Natl Cancer Inst. 2022;114:1577-1583. 36130287 10.1093/jnci/djac135 PMC9745434

[13] Goulart BHL , Unger JM, Chennupati S, Fedorenko CR, Ramsey SD. Out-of-pocket costs for tyrosine kinase inhibitors and patient outcomes in EGFR- and ALK-positive advanced non–small-cell lung cancer. J Clin Oncol Oncol Pract. 2021;17:e130-e139. 10.1200/OP.20.00692 PMC8257903 33284732

[14] Hsu ML , Boulanger MC, Olson S, et al Unmet needs, quality of life, and financial toxicity among survivors of lung cancer. JAMA Netw. Open. 2024;7:e246872. 38630475 10.1001/jamanetworkopen.2024.6872 PMC11024770

[15] Hastert TA , McDougall JA, Strayhorn SM, et al Social needs and health-related quality of life among African American cancer survivors: results from the Detroit Research on Cancer Survivors study. Cancer. 2021;127:467-475. 33225460 10.1002/cncr.33286 PMC7992904

[16] Johnson PC , Markovitz NH, Gray TF, et al Association of social support with overall survival and healthcare utilization in patients with aggressive hematologic malignancies. J Natl Compr Canc Netw. 2021;1:1-7. 10.6004/jnccn.2021.7033 34653964

[17] Health Leads. The health leads screening toolkit. Health Leads; 2022. Accessed June 15, 2024. https://healthleadsusa.org/news-resources/the-health-leads-screening-toolkit/

[18] The PRAPARE screening tool | PRAPARE. Protocol for Responding to and Assessing Patients’ Assets, Risks, and Experiences. Accessed June 15, 2024. https://prapare.org/the-prapare-screening-tool/

[19] Billioux A , Verlander K, Anthony S, Alley D. Standardized screening for health-related social needs in clinical settings: the accountable health communities screening tool. Expert Voices Health Health Care Natl Acad Med. 2017:1.

[20] Haines E , Shelton RC, Foley K, et al Addressing social needs in oncology care: another research-to-practice gap. JNCI Cancer Spectr. 2024;8:pkae032. 38676669 10.1093/jncics/pkae032 PMC11104529

[21] Bernardo BM , Zhang X, Beverly Hery CM, Meadows RJ, Paskett ED. The efficacy and cost-effectiveness of patient navigation programs across the cancer continuum: a systematic review. Cancer. 2019;125:2747-2761. 31034604 10.1002/cncr.32147

[22] Sehgal K , Bulumulle A, Brody H, et al Association of extended dosing intervals or delays in pembrolizumab-based regimens with survival outcomes in advanced non–small-cell lung cancer. Clin. Lung Cancer. 2021;22:e379-e389. 32653295 10.1016/j.cllc.2020.05.028 PMC7273162

[23] Gany F , Melnic I, Wu M, et al Food to overcome outcomes disparities: a randomized controlled trial of food insecurity interventions to improve cancer outcomes. J Clin Oncol. 2022;40:3603-3612. 35709430 10.1200/JCO.21.02400 PMC9622577

[24] Beavis AL , Sanneh A, Stone RL, et al Basic social resource needs screening in the gynecologic oncology clinic: a quality improvement initiative. Am J Obstet Gynecol. 2020;223:735.e1-735.e14. 10.1016/j.ajog.2020.05.028 PMC8340269 32433998

[25] Neparidze N , Godara A, Lin D, et al Impact of social needs and identity experiences on the burden of illness in patients with multiple myeloma: a mixed-methods study. Healthcare. 2024;12:1660. 39201218 10.3390/healthcare12161660 PMC11353550

[26] Patel MI , Khateeb S, Coker T. Association of a lay health worker-led intervention on goals of care, quality of life, and clinical trial participation among low-income and minority adults with cancer. J Clin Oncol Oncol. Pract. 2021;17:e1753-e1762. 10.1200/OP.21.00100 PMC9810146 33999691

[27] Suh E , Owen ED, Reichek J, et al “Getting to know you and your child” screening questionnaire: Results from a Chicago pediatric collaborative. J Clin Oncol. 2018;36:e22515.

[28] Thom B , Aviki EM, Lapen K, Thompson T, Chino F. Screening for health-related social needs and financial toxicity among patients with cancer treated with radiation therapy: findings from a quality improvement project. J Am Coll Radiol JACR. 2024;21:1352-1361. 38971414 10.1016/j.jacr.2024.07.001

[29] Davis J , Zinck L, Kelly S, et al Screened social risk factors & screening acceptability among oncology patients in Philadelphia. J Clin Oncol. 2021;39:12125-12125.

[30] Mazor MB , Li L, Morillo J, et al Disparities in supportive care needs over time between racial and ethnic minority and non-minority patients with advanced lung cancer. J. Pain Symptom Manage. 2022;63:563-571. 35031503 10.1016/j.jpainsymman.2021.12.007 PMC9336182

[31] Warnecke RB , Oh A, Breen N, et al Approaching health disparities from a population perspective: the national institutes of health centers for population health and health disparities. Am J Public Health. 2008;98:1608-1615. 18633099 10.2105/AJPH.2006.102525 PMC2509592

[32] Schoenberger H , Rich NE, Jones P, et al; Multi-Ethnic HCC Cohort Investigators. Racial and ethnic disparities in barriers to care in patients with hepatocellular carcinoma. Clin Gastroenterol Hepatol 2023;21:1094-1096.e2. 34965448 10.1016/j.cgh.2021.12.027 PMC9233716

[33] Oyedele NK , Lansey DG, Chiew C, et al Development and testing of a mobile app to collect social determinants of health data in cancer settings: interview study. JMIR Form Res. 2023;7:e48737. 37707880 10.2196/48737 PMC10540013

[34] Wethington SL , Rositch AF, Yu R, et al Integrating social needs screening and resource referral into standard ambulatory oncology care: a quality improvement project. J Clin Oncol Oncol. Pract. 2024;20:566-571. 10.1200/OP.23.00485 38277618

[35] Beauchemin MP , Solomon S, Michaels CL, et al Toward identification and intervention to address financial toxicity and unmet health‐related social needs among adolescents and emerging adults with cancer and their caregivers: a cross‐cultural perspective. Cancer Med. 2024;13:e7197. 38659403 10.1002/cam4.7197 PMC11043682

[36] Nyakudarika NC , Holschneider CH, Sinno AK. Universal social needs assessment in gynecologic oncology: an important step toward more informed and targeted care in the public safety net. Cancer. 2021;127:3809-3816. 34250590 10.1002/cncr.33761

[37] Leukemia and lymphoma society. LLS community guide: top menu navigation. Accessed June 15, 2024. https://www.lls.org/article/lls-community-guide-top-menu-navigation.

[38] Fouad MN , Acemgil A, Bae S, et al Patient navigation as a model to increase participation of African Americans in cancer clinical trials. J Oncol Pract 2016;12:556-563. 27189356 10.1200/JOP.2015.008946 PMC4957258

[39] Hatef E , Chang H-Y, Richards TM, et al Development of a social risk score in the electronic health record to identify social needs among underserved populations: retrospective study. JMIR Form Res 2024;8:e54732. 38470477 10.2196/54732 PMC10966439

[40] Sinha S , Garriga M, Naik N, et al Disparities in electronic health record patient portal enrollment among oncology patients. JAMA Oncol. 2021;7:935-937. 33830178 10.1001/jamaoncol.2021.0540 PMC8033503

[41] Proctor EK , Bunger AC, Lengnick-Hall R, et al Ten years of implementation outcomes research: a scoping review. Implement Sci. 2023;18:31. 37491242 10.1186/s13012-023-01286-z PMC10367273

[42] Kronfli D , Savla B, Lievers A, et al Identifying psychosocial needs of patients with cancer undergoing curative radiation therapy in an inner-city academic center to address racial disparities. Int J Radiat Oncol Biol Phys. 2022;114:185-194. 35490990 10.1016/j.ijrobp.2022.04.003

[43] Baughman A , Clark MA, Boehmer U. Experiences and concerns of lesbian, gay, or bisexual survivors of colorectal cancer. Oncol Nurs Forum. 2017;44:350-357. 28635972 10.1188/17.ONF.350-357 PMC5920528

[44] Coughlin SS , Ayyala DN, Stewart JL, Cortes JE. Social needs and health-related quality of life among hematologic cancer survivors. Support Care Cancer. 2022;30:8919-8925. 35895158 10.1007/s00520-022-07281-2

[45] Cotangco K , Pineda E, Hingarh V, et al Integrating social care into gynecologic oncology: Identifying and addressing patient’s social needs. Gynecol Oncol. 2023;179:138-144. 37980768 10.1016/j.ygyno.2023.11.001 PMC11218889

[46] Journal of *AHIMA*. New SDOH reporting requirements expected to impact HI workflow, staffing. 2024. Accessed June 15, 2024. https://journal.ahima.org/page/new-sdoh-reporting-requirements-expected-to-impact-hi-workflow-staffing.

[47] Reynolds A. Social need: new HEDIS measure uses electronic data to look at screening, intervention. *NCQA.* 2022. https://www.ncqa.org/blog/social-need-new-hedis-measure-uses-electronic-data-to-look-at-screening-intervention/

[48] Neeman E , Lyon L, Sun H, et al Future of teleoncology: trends and disparities in telehealth and secure message utilization in the COVID-19 Era. J Clin Oncol Clin Cancer Inform 2022;6:e2100160. 10.1200/CCI.21.00160 PMC9067360 35467963

